# Lectin- and Saccharide-Functionalized Nano-Chemiresistor Arrays for Detection and Identification of Pathogenic Bacteria Infection

**DOI:** 10.3390/bios8030063

**Published:** 2018-06-29

**Authors:** Nuvia M. Saucedo, Yingning Gao, Tung Pham, Ashok Mulchandani

**Affiliations:** 1 Department of Chemistry, University of California, Riverside, CA 92521, USA; nsaucedo@southern.edu; 2 Department of Chemistry, Southern Adventist University, Collegedale, TN 37315, USA; 3 Department of Chemical and Environmental Engineering, University of California, Riverside, CA 92521, USA; gaoyingning@gmail.com (Y.G.); tpham052@ucr.edu (T.P.); 4 Materials Science and Engineering Program, University of California, Riverside, CA 92521, USA

**Keywords:** pathogens, lectins, CNTs, PCA, point-of-care, label-free detection

## Abstract

Improvement upon, and expansion of, diagnostic tools for clinical infections have been increasing in recent years. The simplicity and rapidity of techniques are imperative for their adoption and widespread usage at point-of-care. The fabrication and evaluation of such a device is reported in this work. The use of a small bioreceptor array (based on lectin-carbohydrate binding) resulted in a unique response profile, which has the potential to be used for pathogen identification, as demonstrated by Principal Component Analysis (PCA). The performance of the chemiresistive device was tested with *Escherichia coli K12*, *Enterococcus faecalis*, *Streptococcus mutans*, and *Salmonella typhi*. The limits of detection, based on concanavalin A (conA) lectin as the bioreceptor, are 4.7 × 10^3^ cfu/mL, 25 cfu/mL, 7.4 × 10^4^ cfu/mL, and 6.3 × 10^2^ cfu/mL. This shows that the detection of pathogenic bacteria is achieved with clinically relevant concentrations. Importantly, responses measured in spiked artificial saliva showed minimal matrix interference. Furthermore, the exploitation of the distinctive outer composition of the bacteria and selectivity of lectin-carbohydrate interactions allowed for the discrimination of bacterial infections from viral infections, which is a current and urgent need for diagnosing common clinical infections.

## 1. Introduction

The detection of bacteria is important for human health, both to prevent and determine the cause of illness. Recent reports by the Centers for Disease Control and Prevention (CDC) [1] approximate that 48 million cases of infections occur each year in the United States. In the same report, *Salmonella* and *E. coli* ranked number one and five, respectively, with respect to the leading causes of bacterial foodborne illness resulting in hospitalization. While some strains of bacteria have less severe symptoms, exposure to certain strains can lead to serious complications. Such is the case for the *E. coli* strain, O157:H7, whose initial symptoms can progress from diarrhea, fever, and/or urinary tract infections (UTIs) to hemolytic uremic syndrome (HUS) [2], a potentially life-threatening condition. Rapid detection and diagnosis is imperative when dealing with severe and fast-acting bacterial infections and is the first step towards the treatment and recovery of patients. An accurate, yet rapid and simple, sensor would be beneficial, not only in cases of severe infections, but also in diagnosing common infections that occur in the ear, throat, and sinus. Currently, infections rank among the top 10 most common medical misdiagnosis and top 10 causes of death [3]. Development and employment of sensors that can determine if an infection is viral or bacterial would contribute enormously to curbing misdiagnosis and antibiotic misuse [4,5,6]. Additional benefits of developing detection technologies include shorter infection duration and decreased numbers of doctor visits. In this work, we report an array biosensor, which has a fast response time, is easy to use and has the potential to distinguish bacterial from viral infections.

Current methods for the detection of pathogenic bacteria focus on the use of antibodies or DNA. The standard methods are enzyme-linked immunosorbent assay (ELISA) and polymerase chain reaction (PCR) [7], respectively. Generally speaking, ELISA requires ~10^3^ cfu/mL of bacteria [8,9], and the PCR cell requirement varies in the tens of cells to generate a good signal, depending on the target [9]. Though these methods have a high sensitivity and specificity, they require trained personnel and have long assay-times. These limitations impede their use as rapid, broad-range detection platforms, which are critically needed for point-of-care in clinical settings. Many efforts are being made to develop additional techniques for the detection of pathogens, such as quartz crystal microbalance (QCM) [10], surface plasmon resonance (SPR) [11], and optical fiber-based devices [11]. Their sensitivities also depend on the target, and range between 10^2^ and 10^3^ cfu/mL. Some methods, such as culture-based, colorimetric [12], and electrochemical detection [13], can monitor the growth of bacteria cells. This allows for the assessment of the viability of cells over time and after antibiotic treatment. Developing and optimizing growth conditions for new bacteria, though interesting and relatively simple, is time-consuming and material-intensive. Improvements upon culturing methods exemplify the advances in the field of pathogen detection and demonstrates the interest and concern for addressing the lack of point-of-care diagnostics. Electrochemical methods, however, due to their robustness, rapidity, and simplicity, provide a more than suitable and desirable platform for the detection of pathogens [14].

The biosensor reported in this work is able to detect a broad spectrum of bacterial species and, inherently, by use of an array, generates a response profile, which can be used to identify the bacteria without having to modify the device for exquisite selectivity. Briefly, the design of the biosensor began with the development of a carbon nanotube (CNT)-based chemiresistive device. Choosing an electrical method for detection provides quick and simple measurement options, which are easily interpreted. Two types of bioreceptor, lectins and saccharides were used to capture bacterial cells on the device surface, regardless of the strain, which proved to be selective enough to generate a unique response pattern when used in an array format. The former captures bacteria through their polysaccharides, each type of lectin binding saccharides with different affinities [15,16]. The latter captures cells through the lectins existing on the cell surface. Thus, using lectins as receptors and lectins of bacteria as target, it is possible, as is demonstrated in this work, to generate a binding profile for each bacteria. There are only a few reports providing information on these lectins [17,18]. However, the performances of detection schemes were evaluated, compared, and used to compose arrays that result in unique profiles for identifying bacteria.

**Figure 1:**
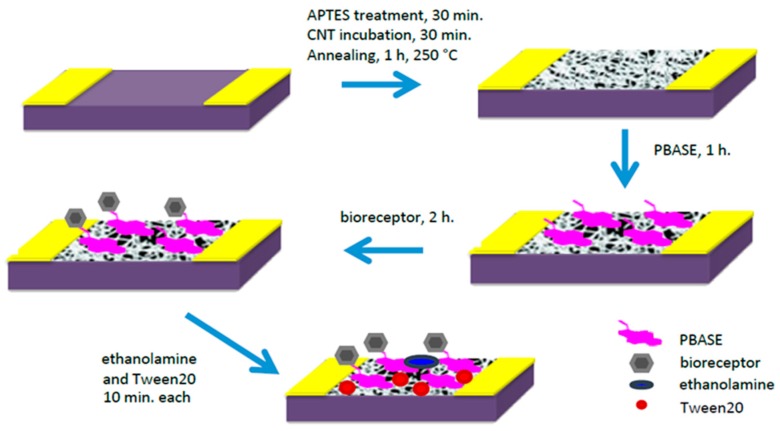
Device fabrication schematic. Using PBASE linker molecule, two bioreceptors can be functionalized onto the surface, amine-modified saccharides or lectin proteins.

In order to functionalize the device with lectin, the CNTs were first incubated with a linker molecule, 1-pyrenebutanoic acid succinimidyl ester (PBASE), so as to not disturb the pristineness/electronic properties of the CNT network, which introduces an amine reactive ester. Upon the addition of the lectins to the PBASE-modified CNTs, the lectins were covalently bound to the surface (Figure 1). Correspondingly, to functionalize them with saccharides, PBASE-modified CNTs were incubated with amine-modified saccharides that bound the saccharides covalently.

## 2. Materials and Methods

### 2.1. Materials

Single-walled carbon nanotubes (SWNTs) were purchased from NanoIntegris (Boisbriand, QC, Canada). To extend their shelf-life, SWNT stock was aliquoted and stored in the dark. 1-pyrenebutanoic acid succinimidyl ester (PBASE, 95%), aminophenyl glucoside (Glc), aminophenyl galactoside (Gal), and wheat-germ agglutinin (WGA) were purchased from Sigma Aldrich (St. Louis, MO, USA) and used as received. Aminophenyl mannoside was purchased from Santa Ana Biotechnology (Santa Ana, CA, USA). Lyophilized lectins, concanavalin A (conA) and arachis hypogeae (arachis) were purchased from MP Biomedicals (Santa Ana, CA, USA) and BioWorld (Dublin, OH, USA), respectively. Ethanolamine (99%) was purchased from Acros Organics (Pittsburgh, PA, USA). Polyethylene glycol sorbitan monolaurate (Tween20) was purchased from BioRad Laboratories (Irvine, CA, USA). Dimethylformamide (DMF) was purchased from Fisher Scientific (Waltham, MA, USA). 10 mM phosphate buffer (PB) was prepared with mono- and di-basic phosphate, purchased from Fisher Scientific, and pH was adjusted to 7.4 using diluted hydrochloric acid (HCl) or diluted sodium hydroxide (NaOH), also purchased from Fisher Scientific. PB was enriched with CaCl_2_ and MnCl_2_, purchased from Fisher Scientific, for lectin and bacteria incubations. The divalent cations (Ca^2+^ and Mn^2+^) must be present and bound to the lectin in order for the binding to occur [15]. All other chemicals and broth components for bacteria culturing were purchased from Fisher Scientific.

### 2.2. Preparation of Functionalized SWNT Devices

**Figure 2:**
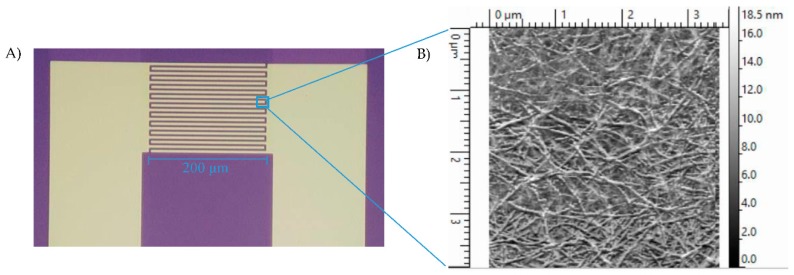
(**A**) Interdigitated gold electrodes on Si/SiO_2_ with twenty 200 µm long and 5 µm wide digits, separated by 3 µm gaps. (**B**) AFM image of CNT-functionalized gap of interdigitated electrodes. Method results in reproducible, dense and uniform surface coverage.

**Figure 3:**
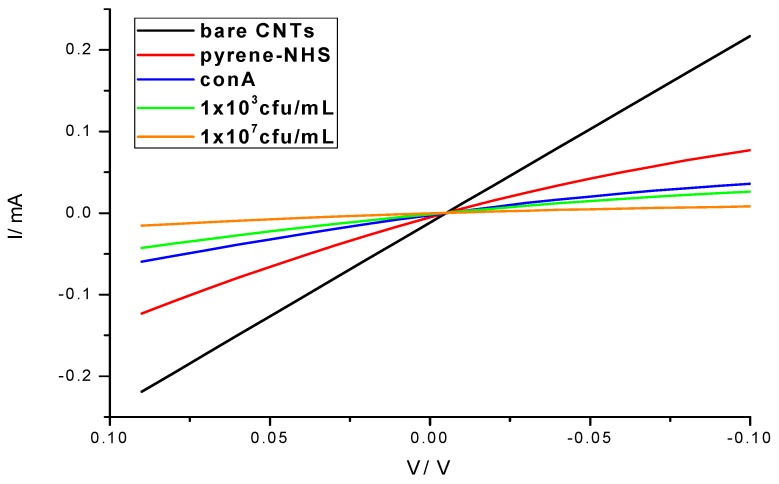
I-V characteristics of the biosensor showing the progression of the device fabrication, surface functionalization and *E. coli* bacteria detection (1 × 10^3^ and 1 × 10^7^ cfu/mL).

The preparation of SWNT devices follows a previously reported method [19]. A schematic of the device preparation is provided in Figure 1. Briefly, the 3 µm-gap-gold electrodes (Figure 2A) were photolithographically patterned onto silicon/silicon dioxide wafers. The surface of these wafers were cleaned by 10 min of sonication in acetone and 30 min in piranha, oxygen plasma and UV ozone, then treated with 1-aminopropyltriethoxy silane (APTES), and incubated with SWNTs, to achieve a uniform assembly of SWNTs on the electrode surface. Excess SWNTs were washed off under running deionized water and the devices were annealed in a Lindberg Blue M tube furnace for 1 h at 250 °C to improve the contact between SWNTs and gold electrodes by removing solvents. As seen in the atomic force microscope (AFM) image (Figure 2B), the device fabrication method used in this work produced uniformly covered dense networks of SWNTs. Devices were incubated with the linker molecule, PBASE (3 mg/mL in DMF), for 1 h, followed by rinsing with DMF and PB. The bioreceptor was then introduced onto the surface of the devices. One of two schemes was used: (a) aminophenyl saccharides (3 mg/mL in DMF) or (b) lectins (3 mg/mL in enriched PB). After 2 h of incubation, the device was rinsed with DMF and PB in the case of aminophenyl saccharides and rinsed with only PB in case of lectins to remove excess. Ethanolamine (0.1% in deionized water) was used to block unreacted PBASE sites and 0.1% Tween20, used to block open SWNT areas, minimizing non-specific binding. The latter two incubations lasted 10 min and were followed by rinsing with PB. To monitor the effectiveness and magnitude of each subsequent functionalization, I-V measurements were performed before and after each incubation step using a CHI 1202A electrochemical workstation, and resistances were calculated from the slope (Figure 3). The drive current in the CNT chemiresistive device at a given voltage decreased after modification with PBASE and a bioreceptor. It is worth noting that the resistance of the device increases with the functionalization steps (Figure 3). This observation is attributed to the addition of negative charge via the π–π stacking interaction between CNTs and pyrene, in the case of PBASE, and the scattering potential generated and/or the electron donation from the molecules to the nanotubes, in the case of bioreceptor [19]. The current remained constant after ethanolamine and Tween20 blocking.

### 2.3. Bacteria Growth

*E. coli* and *Salmonella* were grown in Luria Broth at 37 °C with shaking at 150 rpm, and were harvested during their exponential growth phase, which occurred after 6–9 h. *Enterococcus* and *Streptococcus* were grown in brain heart infusion (BHI) growth media at 37 °C with no shaking, and were harvested during their exponential growth phase, which occurred 6–9 h later. Bacteria were freshly grown on each day of testing. The optical density was measured at 600 nm wavelength on a Beckman Coulter DU800 spectrophotometer to determine the concentration of the bacteria. Serial dilution was carried out with enriched PB to obtain desired concentrations.

### 2.4. MS2 Phage and Influenza H1N1 Virus Preparation

The bacteriophage MS2 and H1N1 virus served as models to test the ability of the device to distinguish between viral and bacterial infections. The bacteriophage MS2 was harvested via the Single Agar Layer (SAL) Environmental Protection Agency (EPA) method 1602. H1N1 virus was propagated in Madin Darby canine kidney (MDCK) cells. Serial dilutions were prepared in PB. Detection of the virus was performed on both lectin and saccharide platforms and followed the same methodology as the bacteria detection, described in Section 2.6.

### 2.5. Artificial Matrices Preparation

Two artificial matrices were prepared: Urine and saliva. Preparation of artificial urine included the following: 170 mM urea, 90 mM KCl, 90 mM NaCl, 25 mM NH_4_Cl, 7.0 mM creatinine, 7.0 mM K_2_HPO_4_, 7.0 mM KH_2_PO_4_, 2.5 mM CaCl_2_, 2.0 mM MgSO_4_, 2.0 mM MnCl_2_, and 0.4 mM uric acid mixed together in deionized water with pH adjusted to 6.25, by the addition of HCl, and refrigerated at 4 °C [20]. Preparation of artificial saliva included the following: 2 g/L mucin, 50 mM urea, 6.8 mM NaCl, 5.4 mM CaCl_2_, 5.4 mM KCl, 4.2 mM Na_2_HPO_4_, and 2.0 mM MnCl_2_ dissolved in deionized water, adjusted to pH 7.2 with HCl, sterilized by autoclaving and stored in the refrigerator until use [21]. Both artificial matrices were used within 2 weeks of preparation. Artificial matrices were spiked with bacteria, followed by serial dilutions using the respective matrix.

### 2.6. Detection

The lectin-functionalized CNT devices were incubated with bacteria for 1 h at room temperature. The unbound bacteria were washed away with PB. The addition of charge, as stated earlier, results in a change in the device’s electronic properties. The negatively charged bacteria produces a change in these properties which can be monitored by measuring device resistance [19]. The resistance of the device at each concentration was measured by scanning the potential between the source and drain electrodes from −0.2 V to +0.2 V at a scan rate of 20 mV/s, and recording the current using a CHI 1202A electrochemical workstation. The resistance is the inverse of the slope of the I-V curve.

## 3. Results and Discussion

### 3.1. Device Reproducibility

The electrodes prepared by the afore-mentioned process displayed very good reproducibility in initial resistance, stability, and performance. Intra-chip resistances (55 electrodes) were within 19% of each other. Stability studies on 25 electrodes over a one-month period retained 103% ± 12% of their initial resistance.

### 3.2. Validation Studies

Initially, lectins having high or no specificity to aminophenyl saccharides were tested to demonstrate the ability of our device to bind bacteria through their naturally occurring surface lectins. The lectins used in this work were conA, arachis, and WGA. Literature on lectin and saccharide affinities report that conA has the highest affinity to glucose and mannose, arachis to galactose and WGA to *N*-acetylglucosamine [22,23]. Stock solutions of each lectin (3 mg/mL) were made fresh for each set of trials and prepared in enriched PB (containing 1 mM Ca^2+^ and Mn^2+^ ions). These ions are required for specific lectins, called c-lectins, which have metal binding sites that must be occupied in order to attain correct conformation allowing them to bind carbohydrates [22].

**Figure 4:**
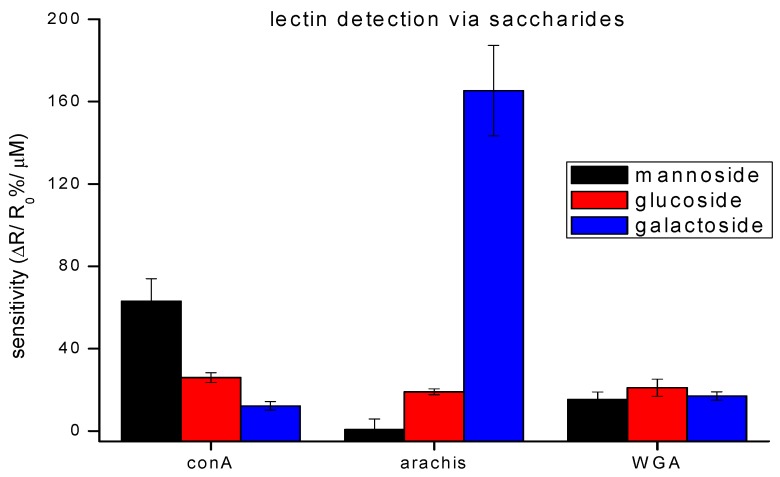
Detection of different lectins on a saccharide-functionalized CNT-device. The bar graphs show a relative binding affinity with saccharides for three lectins. The data points are average measurements from 5 independent biosensors, and error bars represent ±1 standard deviation.

Validation of protocols and incubation methods, through the detection of free lectins by their respective saccharides, shows good correlation to literature reports (Figure 4) [23,24]. While each lectin has a saccharide to which it binds most strongly, the strength of that highest affinity is different. The affinity binding constants attest to this [25,26,27]. The detection scheme was designed to take advantage of these differences. For example, the detection of WGA, captured through galactoside, towards which it has low affinity, resulted in a small signal. Whereas, detection of arachis via galactoside, towards which it has a high affinity, provided a large signal. For these reasons, the signals of lectin detection via their respective saccharides vary.

### 3.3. Device Sensitivity

**Figure 5:**
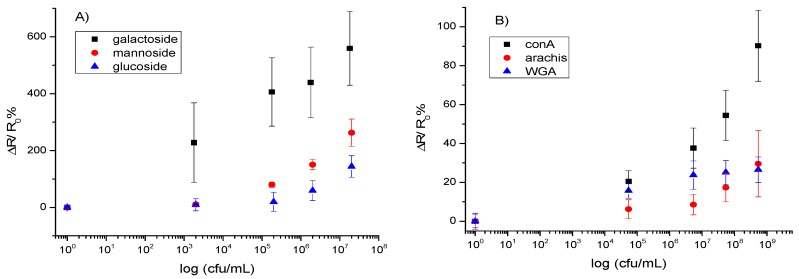
Calibration curves for the detection of (**A**) *E. coli* via saccharide-modified CNT-devices and (**B**) *Streptococcus mutans* via lectin-modified CNT-devices. The data points are average measurements from 10 independent biosensors, and error bars represent one standard deviation.

As stated above, PBASE can react with amine-modified saccharides which in turn can capture bacteria through their surface lectins. Devices functionalized with aminophenyl saccharides were each incubated with one of the four different bacteria used in this work at varying concentrations for 1 h, followed by rinsing with PB and resistance measurement for the quantitative detection, sensitivity, dynamic range, and limit of detection. The normalized response of the biosensor [(*R* − *R*_0_)/*R*_0_], where *R*_0_ is the resistance of the device after functionalization and blocking of unoccupied sites with Tween20, and *R* is the resistance after incubation with bacteria] as a function of the log of bacteria concentration is shown in Figure 5A. This treatment of the data showed the clearest relationship between the target and receptor. As shown in Figure 5A, the response is correlated linearly to the log of concentration over the detection range, the slope of which differs for each target/bioreceptor combination used. This agrees with other studies of similar systems [11,28]. Regression equations fitted for the concentration range, 2 × 10^3^ to 2 × 10^8^ cfu/mL, were: *y* = 0.92*x* − 0.90 (*R*^2^ = 0.95) for *E. coli* on galactoside, *y* = 0.61*x* − 2.2 (*R*^2^ = 0.92) for *E. coli* on mannoside, and *y* = 0.31*x* − 1.1 (*R*^2^ = 0.62) for *E. coli* on glucoside, where *y* is the relative change in resistance [(*R* − *R*_0_)/*R*_0_] and *x* is the logarithmic concentration of bacteria.

Alternatively, PBASE can react with the amines found on the lectins. This in turn targets the saccharides on the surface of the bacteria for binding.

Figure 5B shows the normalized response [(*R* − *R*_0_)/*R*_0_] of the biosensor. As shown in the figure, the response is linear over the detection range, which differs for each bioreceptor used. Regression equations, fitted from 5.5 × 10^4^ to 5.5 × 10^8^ cfu/mL, were: *y* = 0.16*x* − 0.64 (*R*^2^ = 0.82) for Streptococcus on conA, *y* = 0.027*x* + 0.037 (*R*^2^ = 0.90) for Streptococcus on WGA, and *y* = 0.056*x* − 0.23 (*R*^2^ = 0.71) for *Streptococcus* on arachis, where *y* is the relative change in resistance [(*R* − *R*_0_)/*R*_0_] and *x* is the logarithmic concentration of bacteria. 

When detecting bacteria in relevant artificial matrices, several challenges were faced. For the detection of *E. coli* in artificial urine, detection was suppressed 10-fold by the presence of urea. It is known that urea, at high enough concentrations, can unfold proteins [29]. For the detection scheme employed here, unfolding the protein means losing the binding pocket and therefore losing detection. The device could be regenerated by, first, treating the surface with a diluted HCl and glycine mixture [26] to disrupt the interaction between the cells and receptor (unbinding them from the surface) and then rinsing it with enriched PB solution to return the lectin to the correct binding conformation.

In the case of *Streptococcus mutans*, the mucin in the artificial saliva contributed some background signal. To minimize the adhesion of mucin to the device, the CNT-functionalized device was treated with 0.1% mercaptohexanol (MCH) prior to subsequent functionalization steps [30]. The contribution of the mucin after MCH treatment was determined to be 20% of the signal, a result similar to that obtained in previously reported work [30].

### 3.4. Identification of Infection-Type

#### 3.4.1. Response Profiles

**Figure 6:**
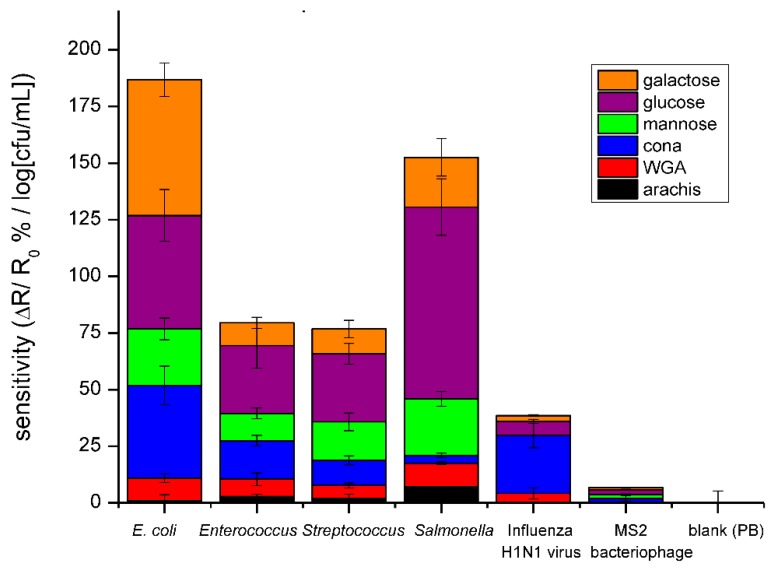
Bar graph comparing the average sensitivities for detection of *E. coli*, *Enterococcus faecalis*, *Enterococcus mutans*, *Salmonella*, H1N1 Influenza virus, and M13 bacteriophage in PB using the three lectins and saccharides. The data points are average measurements from 10 independent biosensors, and the error bars represent ±1 standard deviation.

Detection of four bacteria, one bacteriophage and one virus was achieved in a phosphate buffer on the same three lectins and three saccharides used in the method validation. These results can be seen in Figure 6, in which the slopes of fitted calibration curves, Δ*R*/*R*_0_%/log cfu/mL, are reported. The bacteriophage had little affinity with any of the 6 receptors, while the H1N1 virus had a slight affinity with each receptor, most notably with the lectins. This is corroborated by previous work, which reports virus separation from matrix through agglutination via lectins [27]. The low response of device to viruses is twofold. The small size of the virus contributes less to the electronic properties of device and their surface lacks the targeted molecules. However, detection of bacteria by these six receptors resulted in much larger responses and unique profiles for each bacteria. Because Gram-negative bacteria have a lipopolysaccharide (LPS) layer, which has a high variation in structure and composition [15], these bacteria produced the most different response patterns and are easily distinguishable. On the other hand, Gram-positive bacteria are similar in their outer structure, which is composed mainly of peptidoglycan. Because they are similar, the two Gram-positive bacteria tested in this work resulted in similar response patterns. It is necessary to note that the responses of saccharide-functionalized devices produced a higher response than the lectins, and this provides a higher sensitivity. Additionally, because not all bacteria have lectins on their surface, using saccharides as the bioreceptor results in a higher specificity. From these results, an array, whose bioreceptors are chosen carefully, could be used to distinguish between bacterial and viral infections, and has the potential to discriminate Gram-type bacteria and, in the case of Gram-positive bacteria, further identify the species.

#### 3.4.2. Principal Component Analysis (PCA)

In order to statistically determine that the responses of each bacteria were unique or differentiable, principal component analysis (PCA) was administered. PCA is a chemometric tool used to analyze multivariable data [31]. Often plotting the resulting PCA scores generates clusters. The proximity of theses clusters can be interpreted as the extent to which the data are related or similar i.e., if generated clusters do not overlap, the data of each cluster are distinguishable or different. Determining likeness of data from each bacteria was the goal of performing this analysis. From the PCA analysis and subsequent plots, one can extract which variables contribute most to, or most define, the uniqueness of the analyte, in this case the bacteria. This is useful for designing an array, which is composed of the strongest or most important receptors for discriminating the bacteria. Therefore, an optimized array with minimal components can be designed to have a high selectivity.

**Figure 7:**
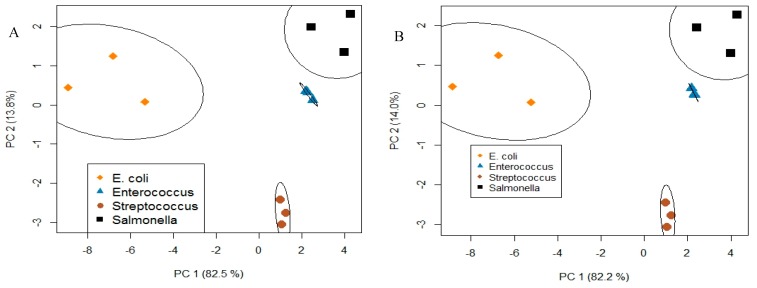
PCA analysis and plot results in clustering with respect to a bacteria type with good resolution using (**A**) six receptors (3 saccharides and 3 lectins) and (**B**) four receptors (3 saccharides and conA lectin). It is shown that a reduced array retains the ability to distinguish four bacteria.

Data, consisting of individual measurements (Δ*R*/*R*_0_) of bacterial concentrations between 2 × 10^3^ and 6 × 10^7^ cfu/mL for 6 receptors, were arranged as an 18-element row. Each row corresponds to a complete array. Three rows were generated for each of the four bacteria. 95% of the total variance across the array was captured by the first two PCA factors. Plotting PCA scores for these two factors (Figure 7A) showed 4 individual clusters, one for each bacterium, demonstrating that each is distinguishable regardless of their Gram-type.

This analysis was repeated using different combinations of the 6 receptors in order to determine which receptors contributed most to the uniqueness of the response profiles. Data from the three saccharides and conA had the tightest clustering among all combinations (Figure 7B). This means that saccharides and conA lectin were the four major contributors to the uniqueness of the response profiles and that four receptors were sufficient to retain cluster separation (i.e., bacteria identification). This method is useful for optimizing arrays to a minimum number, which will improve cost-effectiveness and the overall efficiency of devices. 

## 4. Conclusions

In conclusion, an effective platform to capture bacteria has been developed through the exploitation of lectin-carbohydrate interactions. Results demonstrate the possibility of using this sensor to distinguish between viral and bacterial infections, which is greatly needed for point-of-care applications, and can do so for clinically relevant bacterial concentrations [32]. The resultant response profiles can assist in determining Gram-type, which can guide the selection of antibiotics, another area which relies too heavily on “trial and error” approaches. In the future our work will include the development of similar platforms to distinguish viable and non-viable cells, providing a method for determining the effectiveness of antibiotics against individual infections.
